# Acclimation response and ability of growth and photosynthesis of terrestrial cyanobacterium *Cylindrospermum* sp. strain FS 64 under combined environmental factors

**DOI:** 10.1007/s00203-022-02772-6

**Published:** 2022-02-05

**Authors:** Nadia Bahavar, Shadman Shokravi

**Affiliations:** 1 Plant Physiology Laboratory, Bioscience Faculty, Universidad Autónoma de Barcelona, 08193 Bellaterra, Spain; 2 Department of Biology, Gorgan Branch, Islamic Azad University, Gorgan, Iran

**Keywords:** Alkalinity, Cyanobacteria, *Cylindrospermum* sp. FS 64, Salinity, Time

## Abstract

This investigation tested the hypothesis that the native cyanobacteria can acclimatize and grow under the combination of environmental factors and/or how does their process change with the age of culture? Here, we tried to combine multiple factors to simulated what happens in natural ecosystems. We analyzed the physiological response of terrestrial cyanobacterium, *Cylindrospermum* sp. FS 64 under combination effect of different salinity (17, 80, and 160 mM) and alkaline pHs (9 and 11) at extremely limited carbon dioxide concentration (no aeration) up to 96 h. Our evidence showed that growth, biomass, photosystem II, and phycobilisome activity significantly increased under 80 mM salinity and pH 11. In addition, this combined condition led to a significant increase in maximum light-saturated photosynthesis activity and photosynthetic efficiency. While phycobilisomes and photosystem activity decreased by increasing salinity (160 mM) which caused decreased growth rates after 96 h. The single-cell study (CLMS microscopy) which illustrated the physiological state of the individual and active-cell confirmed the efficiency and effectiveness of both photosystems and phycobilisome under the combined effect of 80 mM salinity and pH 11.

## Introduction

Cyanobacteria are a self-sufficient system that is widely distributed across terrestrial and aquatic environments; and terrestrial cyanobacteria plays a fundamental role in the biological cycle of agriculture (Mareš et al. 2014; Shokravi and Bahavar 2021a). They produce various bio-available elements such as Nitrogen and phosphorus, which are the essential nutrients for plant cultivation (Chittora et al. 2020). Moreover, they generally exhibit a high level of adaptive abilities and tolerance to a large number of environmental factor (Singh 2018). In nature, cyanobacteria are exposed to a constantly changing environment including irradiance, temperature, pH, nutrient availability, salinity, dissolved inorganic carbon fluctuations (Chris et al. 2006; Bouazzara et al. 2020). These changes continuously expose the cyanobacteria cells to multiple stressors of varying magnitude and duration (Borowitzka 2018). In practice, the survival and growth of cyanobacteria depend on their ability to acclimate varying environmental conditions.

Among all cultural parameters, pH is one of the most important factors determining cyanobacteria growth and physiology (Pawlik-Skowrońska et al. 1997; Hinners et al. 2015). Most cyanobacteria have the ability to grow over a wide range of alkaline pH in laboratory (Kaushik 1994). Nearly nothing is known about the mechanisms of cellular survival, cell stability, or growth of cyanobacteria under extreme alkaline conditions (Jangir et al. 2021). Elevated pH (alkalinity) directly influences growth rate and cell yield (de Souza Santos et al. 2011; Shokravi and Bahavar 2021a, b), enzyme activity (Li et al. 2013), biosorption (El-Din 2017), resistance to oxidative stress (Summerfield et al. 2013), and protection (Pathak et al. 2018). It also strongly affects the cyanobacterial abundance (Krausfeldt et al. 2019; Nguyen and Rittmann 2015). While in some cyanobacteria, the growth decreased with the increasing alkalinity (Shokravi and Bahavar 2021a). Therefore, evaluation the effects of different pHs on cyanobacteria is important. In paddy fields, the pH of floodwater varies during the day. Likewise, DIC concentration in the floodwater varies daily and seasonally depending on photosynthetic and respiratory rate (Pedersen et al. 2013). The chemical equilibrium between photosynthesis and respiration implies a balance between inorganic carbon and net ecosystem production (Khan et al. 2020).

Salinity is another environmental factor that could potentially determine the cyanobacteria community in natural ecosystems. Salinity as an essential factor induces diverse alterations in the growth and photosynthesis (Bemal and Anil 2018), biochemical like carbohydrate content (Singh et al. 2015), and physiological characteristics of cyanobacteria (Miriam et al. 2017; Lee et al. 2021). Time (age of the culture) is another essential factor in the resistance and growth in different conditions (Alcorta et al. 2019) which less has been considered (Bouazzara et al. 2020; Jangir et al. 2021). Exposure to initial hours of new condition may create a significant effect on physiological activities during the next hours (Abbasi et al. 2019; Shokravi and Bahavar 2021a). However, there is increasing evidence that the combined environmental factors can be modulated by other factors and led to regulation, acclimation, and adaptation (Müller et al. 1993; Shokravi and Bahavar 2021a). Therefore, studying environmental fluctuations in the short-time regime on cyanobacteria is essential to serve the sustainable development economy in the future.

In the present study, we have selected the filamentous cyanobacterium *Cylindrospermum* sp. for the abundance, fixed Nitrogen, and environmental stability. *Cylindrospermum* sp. FS 64 was isolated from paddy fields of the North of Iran which description is following: aggregations bright blue-green, green–brown, sticky colonies, not regular, expanded; mucilaginous, attached to margin; filaments straight, curved entangled; trichrome 9*µ* broad, constricted at cross wall; cells variable in size, cylindrical or nearly quadrate; heterocyst ovate or ellipsoid, terminal at one side; spore oval, ellipsoidal, adjoining the heterocyst, granular, 10.4*µ* long.

So far, most studies on this genus have been focused on molecular biology (Srivastava et al. 2009; Katoch et al. 2016), physiological characteristics (Briand et al. 2004), proline accumulation (Chris et al. 2006), heavy metal stress (Singh et al. 1989; Chris 2012), Nitrogen forms effect (Kenesi et al. 2009) and chemical analysis (Mareš et al. 2014). The aim of this research is to characterize the acclimation behaviors of *Cylindrospermum* sp. FS 64 under different pH, salinity conditions under extremely limited carbon dioxide concentration for 96 h.

## Materials and methods

### Culture maintenance and growth conditions

*Cylindrospermum* sp. FS 64 was isolated from paddy fields of the North of Iran (Siahbalaei et al. 2011) and collected again by the authors in 2018. The soil samples was serially diluted in sterilized liquid Nitrogen-free medium (BG-110) (Stanier et al. 1971). Isolation was done by streaking and spreading technique on solid BG-110 medium. Purification was done by alternative sub-culturing between liquid and solid BG-110 medium (Shokravi and Bahavar 2021b)*. *The sample was identified and described using multidisciplinary approaches (Molecular 16S rRNA, and morphology using light, fluorescence, and phase-contrast microscopy). Strain after identification as *Cylindrospermum* sp. FS 64 was coded and preserved in the algae museum of the institute of applied sciences of Shahid Beheshti University, Tehran-Iran. The axenic culture were maintained in a liquid BG-110 at temperature 30 ± 2 °C under a constant irradiance of 60 μmol quanta m^−2^ s^−1^ (Poza-Carrión et al. 2001). The pH was adjusted in 7.8 by NaOH.

### Growth conditions and analysis

Growth of *Cylindrospermum* sp. FS 64—in an exponential growth phase—was carried out at various salinity concentrations 17 (culture media without NaCl), 80 and 160 mM at alkaline pHs (9 and 11). Culture media were buffered with 10 mM BTP (Bis–Tris Propane) for pH 9 and 11 adjusted to the desired pH with KOH (Shokravi and Soltani 2011). We studied cultures without CO_2_ or O_2_ bubbling and stirring (standing condition, extremely DIC limitation) (Poza-Carrión et al. 2001; Shokravi and Bahavar 2021a). The determination of the growth was performed using time-course measurements by the correlation between optical density (OD 750 nm), in vivo fluorescence, and counting cells according to Briand et al. (2004) using the CLMS at different salinity and alkaline pHs up to 96 h. The OD was measured using Synergy HTX (Multi-Mode Microplate Reader, USA). Growth rates (*µ*) were calculated according to Li et al. (2014) and Khazi et al. (2018). The absorbance of Chlorophyll content was determined spectrophotometrically at 665 nm according to Marker (1972).

### Physiological characterization

To survey the photosynthetic activity and respiratory electron transport chains under different salinity and alkaline conditions, oxygen exchange was studied. Steady-state oxygen evolution was measured with a Clark-type electrode PSII activity in whole cells. Cells cultured at temperature 30 ± 2 °C and constant illumination 60 μmol quanta m^−2^ s^−1^ (Inoue-Kashino et al. 2005). The amount of liberated oxygen was normalized by the amount of chlorophyll according to Poza-Carrion (Poza-Carrión et al. 2001). The initial physiological status of *Cylindrospermum* sp. FS 64 was performed by measuring the maximum photosynthetic rate (*P*_max_) and photosynthetic efficiency (*α*) and light saturation (*I*_*k*_) values after growth analysis. Photosynthesis–irradiance (*P*–*I*) curves were calculated by measuring oxygen evaluation rates during successive 1-min illumination periods with a stepwise increase from 0 to 2500 μmol quanta m^−2^ s^−1^. The photosynthetic pigments were estimated in terms of chlorophyll a, phycocyanin, from 380 to 760 nm using Synergy HTX (Multi-Mode Microplate Reader, USA) and they normalized to optical density according to Tang and Vincent (1999). The operation of photosystems and phycobilisomes characteristics were analyzed spectrofluorimetrically according to Inou-Kashino et al. (2005), Vermaas et al. (2008) and Zorz et al. (2015). Room temperature fluorescence emission spectra of the cells were recorded following Tiwari and Mohanty (1996) and Fraser et al. (2013). The excitation spectra were recorded at *λ*ex: 440 to excite chlorophyll a and 550 nm for phycocyanin. The single-cell study (the fluorescence intensity of single cell) which illustrated the physiological state of the individual, live-cell and spectral unmixing (Grigoryeva and Chistyakova  2019) was measured using *λ* scan of confocal laser microscope system (Leica TCS-SP5 CLSM—Leica Microsystems Heidelberg GmbH, Mannheim, Germany). Photosynthetic pigment excitation was carried out with an argon laser at 405 nm. The fluorescence emission spectrum was collected by detecting wavelengths between 415 and 760 (Ramírez et al. 2011; Sugiura and Itoh 2012; Shokravi and Bahavar 2021a, b). Analysis of the lambda scan data was carried out using the Leica Confocal Software.

### Statistical analysis

Analysis of variance (ANOVA) with the SPSS-24 software was used to evaluate the results. The ANOVA showed a significant difference between treatments with *p* < 0.05. All the experiments were carried out in six independent biological replicates. For relationships of photosynthesis activity, growth and age of cultures, we fitted a model to the data using interpolation in MATLAB software.

## Results

### Growth

**Fig. 1:**
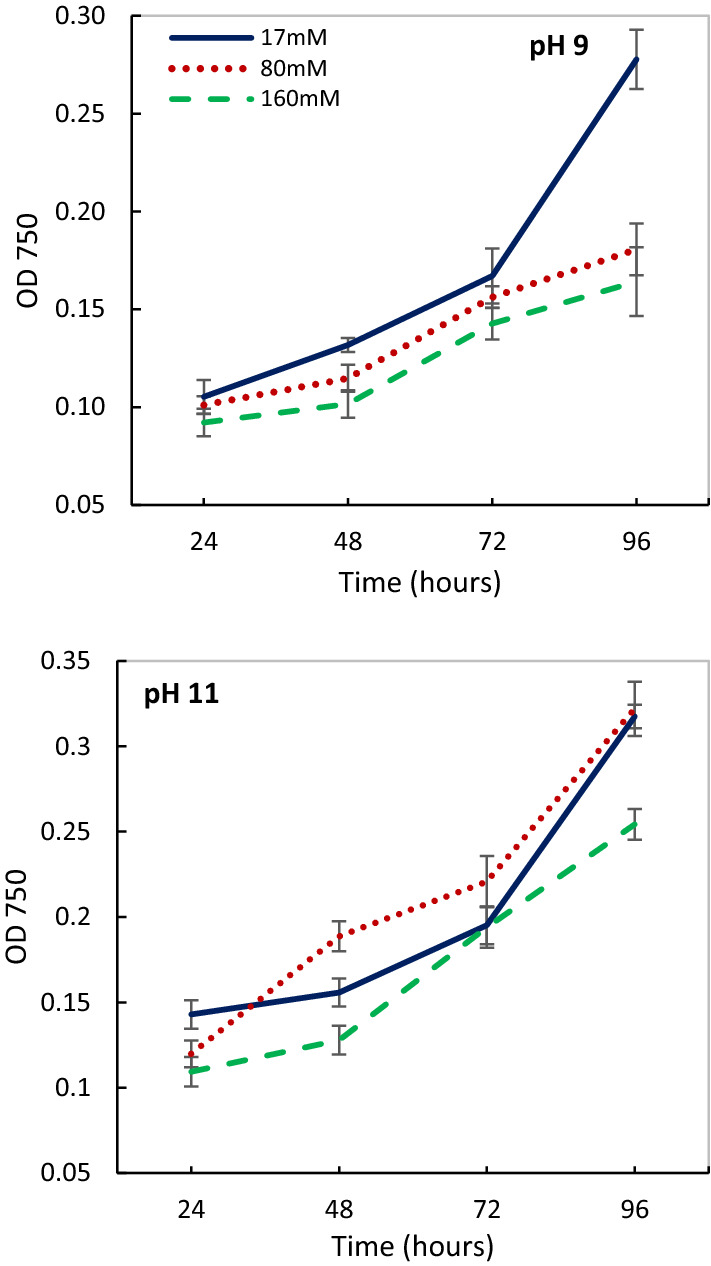
Variation of optical density (OD 750 nm) of *Cylindrospermum* sp. FS 64 under different salinity (17, 80 and 160 mM NaCl) and alkaline pHs (9, 11) from 24 to 96 h. Line with error bars shows significant difference at *P* < 0.05

This study evaluated—in vivo experiments—the ability of acclimation and growth of *Cylindrospermum* sp. FS 64 under multiple environmental factors at a short period of time under extreme DIC limitation. In general, the results supported the hypothesis that the combined environmental factors can be led to growth and acclimation in different environmental conditions at short time. Comparison of the growth curve of *Cylindrospermum* sp. FS 64 showed that extreme alkaline condition (pH 11) was more favorable to growth and had a significant effect (p < 0.05) on biomass production compared to pH 9 under extreme DIC limitation (Fig. 1). A study of the length of the incubation period revealed that 80 mM salinity caused significantly growth increased at pH 11 after 48 h and, likewise no significant effect was observed between 17 and 80 mM salinity at pH 11 after 96 h. Regardless of salinity, pH 11 was the optimum pH in this strain up to 168 h (Result not shown). The presence of 160 mM salinity at both alkaline pHs caused significant inhibition of growth and biomass production until 96 h. The metabolic activities and synthesizing enzymes in the growth stage led to the shorter lag phase under alkaline pHs. Therefore, cells acclimatized in both combined conditions 17 mM—pH 9, and 80 mM—pH 11 after 24 h.

### Photosynthesis (photosynthetic oxygen evolution and *P*–*I* curve)

**Fig. 2:**
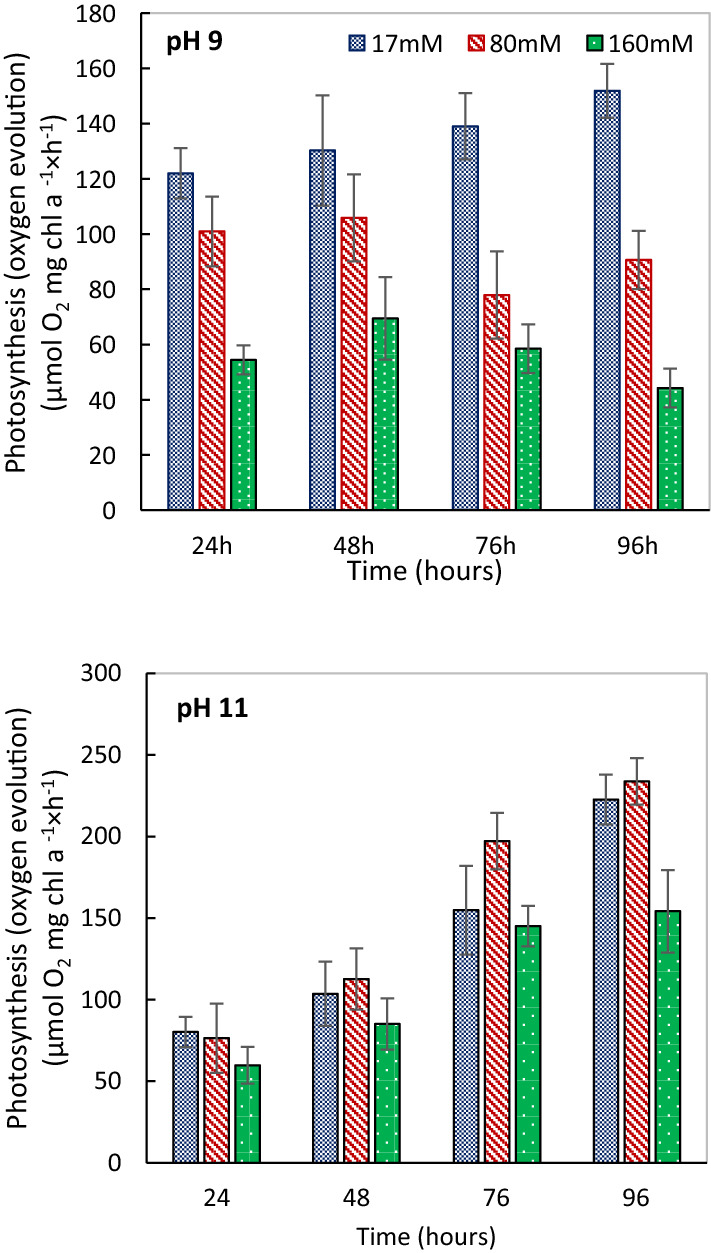
Comparison of photosynthetic oxygen evolution of *Cylindrospermum* sp. FS 64 at different salinity, pHs and time. Bars with error bars show significant difference at *P* < 0.05

**Fig. 3:**
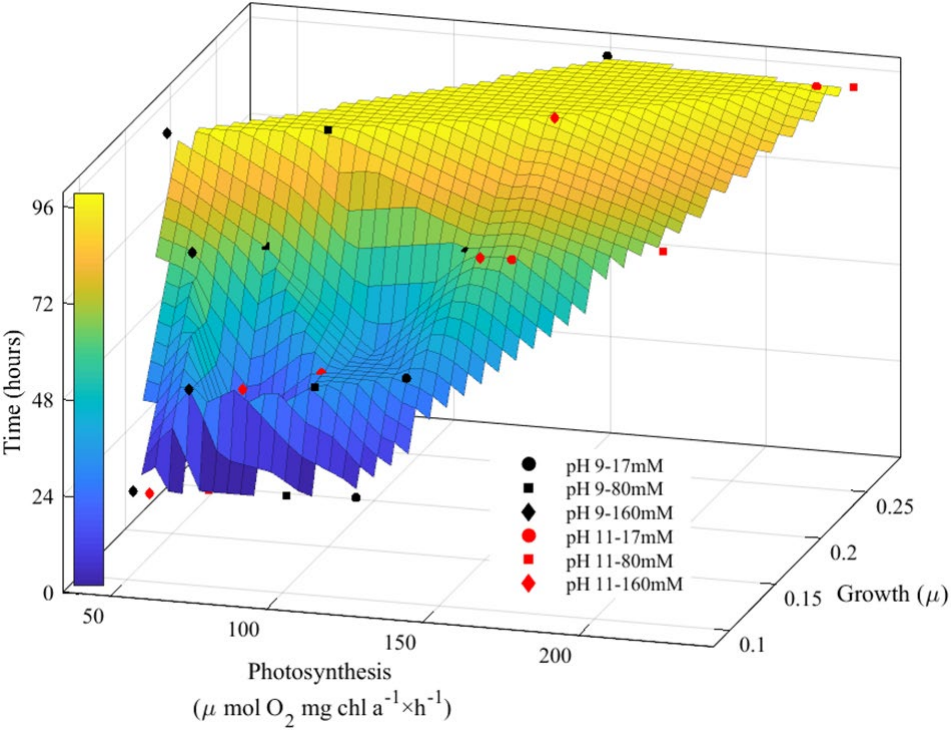
The fitting model of *Cylindrospermum* sp. FS 64 presented the relation between the growth, photosynthesis and time under different levels of salinity and alkalinity treatment. Each data-point represents different salinity and pH

Regardless of salinity and age of the cultures, the maximum rates of oxygen evolution of *Cylindrospermum* sp. FS 64 gradually increased at pH 11 (~ 100–250 µmol O_2_ mg Chl *a*^−1^ × h^−1^) against pH 9 (~ 120–160 µmol O_2_ mg Chl *a*^−1^ × h^−1^) (Fig. 2). The combined effect of salinity, alkalinity and age of culture revealed that the maximum rates of oxygen evolution significantly increased at pH 11 and 80 mM salinity after 48 h (Fig. 2b). In contrast, the significant decrease was observed under high salinity (160 mM) after 72 h at pH 9. Our purpose of the fitting model was to examine the relationships and quantitative description between photosynthesis, growth, and age of culture (Fig. 3). We observed a significant increase in growth and biomass production and photosynthesis activity under the combined environmental factors. A positive and significant correlation was found under the combined 80 mM salinity and pH 11 after 96 h.

The combined effects of environmental factors on photosynthetic parameters (*P*–*I*) are summarized in Table 1. The maximum value of the photosynthesis activity (*P*_max_)—indicating carboxylation or a step closely associated with carboxylation—was approximately 90% higher at combined 80 mM salinity and pH 11 compared to pH 9. Ik indicating the irradiance at which control of photosynthesis passes from light absorption and photochemical energy conversion to reductant utilization (Sakshaug et al. 1997). *I*_*k*_ was lower at the combination of low salinity (17 and 80 mM) and pH 11 compared to pH 9, indicating that the rate of water oxidation in PSII was reduced. *α* is often used for comparing the cyanobacteria shade endurance (shade tendency) under shading conditions. Noticeably, in the presence of 80 mM salinity, the shade-adapted capacity of cyanobacterium significantly increased at both alkaline pHs.

**Table 1:** Comparison of parameters of photosynthesis–irradiance curves (*P*_max_, the maximum photosynthetic rate (μmol O_2_ mg chl^−1^ h^−1^); *α*, photosynthetic efficiency (μmol O_2_ mg chl^−1^ h^−1^)/(μmol photon m^−2^ s^−1^); *I*_*k*,_ light saturation point (µmquanta m^−2^ s^−1^) of *Cylindrospermum* sp. FS 64 at different salinity and alkaline pHs after 72 h of inoculation

NaCl (mM)	17	80	160
*P*_max_—pH 9	67.41 ± 5.55	59.34 ± 4.22	52.45 ± 6.08
*P*_max_—pH 11	54.41 ± 3.05	74.34 ± 6.66	34.45 ± 3.18
*α*—pH 9	0.84 ± 0.04	0.85 ± 0.06	0.61 ± 0.16
*α*—pH 11	0.74 ± 0.02	0.87 ± 0.04	0.65 ± 0.1
*I*_*k*_—pH 9	390	280	520
*I*_*k*_—pH 11	260	120	440

Values are means of three independent biological replicates ± standard deviation

### Absorption spectra

**Fig. 4:**
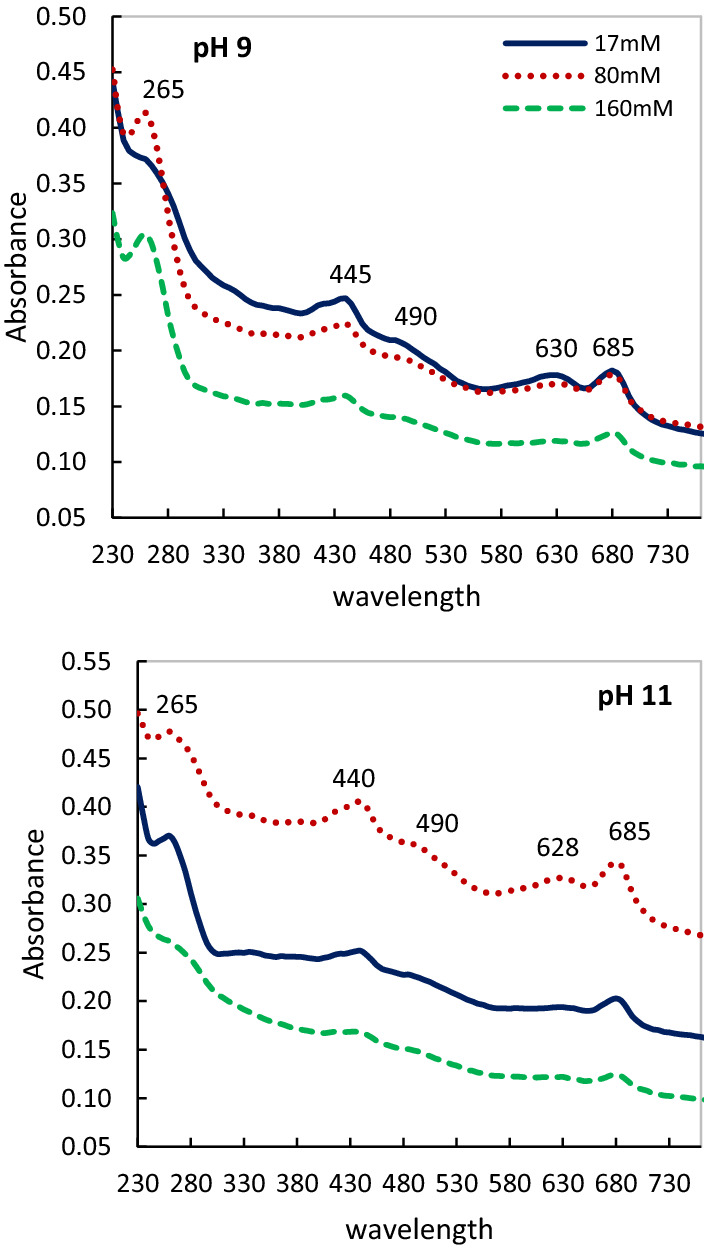
Room temperature absorption spectra of in vivo* Cylindrospermum* sp. FS 64 cells adapted to different salinity and pHs for 72 h. Normalized to optical density (OD 750)

In vivo absorbance spectroscopy as a common method to obtain an overview of the content and distribution of the pigments of cells (Fig. 4) showed that chlorophyll Soret bands (~ 445 nm), chlorophyll a of PSII (~ 680 nm), and phycocyanin (~ 620–630 nm) was the principal active compound in all treatments. A shoulder at ~ 495 nm related to carotenoids has appeared. In addition, we observed an increase in absorption of ~ 250–300 nm which is most probably related to one of the carotenoids bands (generally, organic molecules) under the combination of pH 11 and 80 mM salinity. Our results revealed that the dynamism and stability of PSII and phycobilisomes significantly increase in the combined effect of 80 mM salinity and pH 11 compared to pH 9 after 72 h. While no significant differences were observed between 17 and 80 mM salinity at pH 9. In presence of 160 mM salinity, the structure and stability of phycocyanin and PSII were demolished at pH 11 after 72 h, although they are maintained their structure up under pH 9. In addition, the absence of the shift and stability of the Chl *a* of PSII was noticeable in all treatments.

### PBS and PSII stability under different salinity and alkaline pHs

**Fig. 5:**
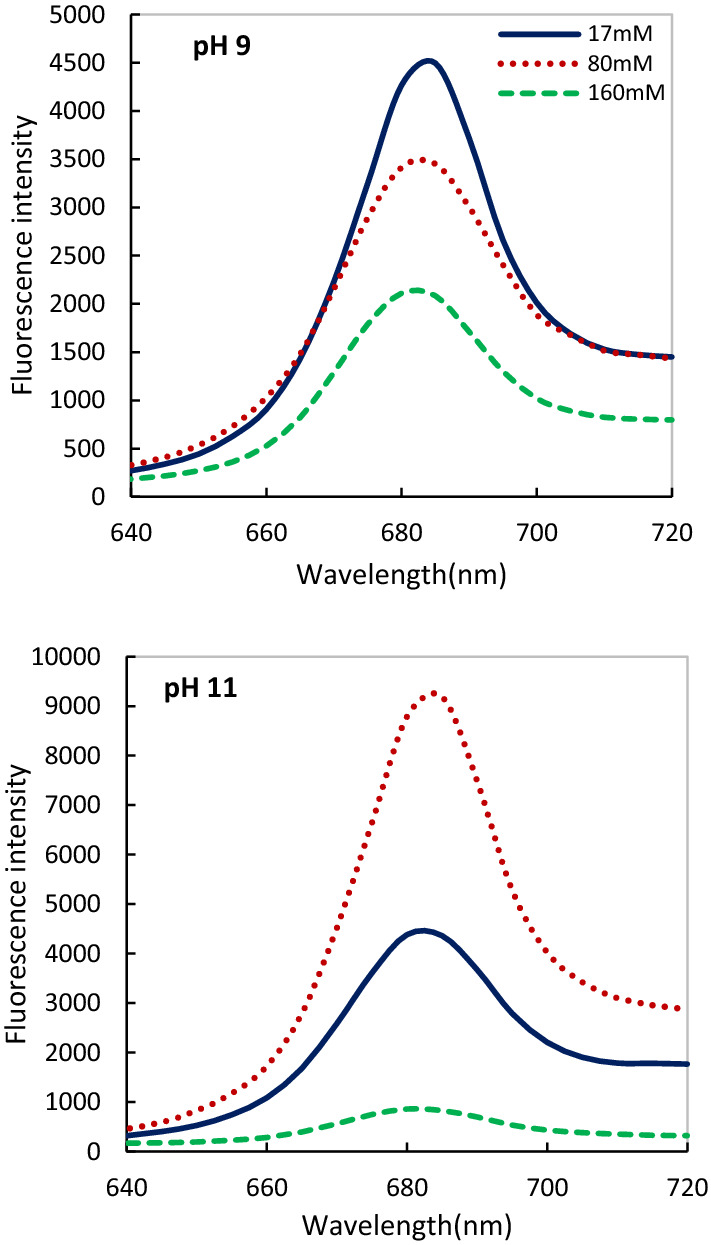
Comparison of the fluorescence intensity of *Cylindrospermum* sp. FS 64 at different salinity and pHs after 72 h of inoculation. Excitation 440 nm

**Fig. 6:**
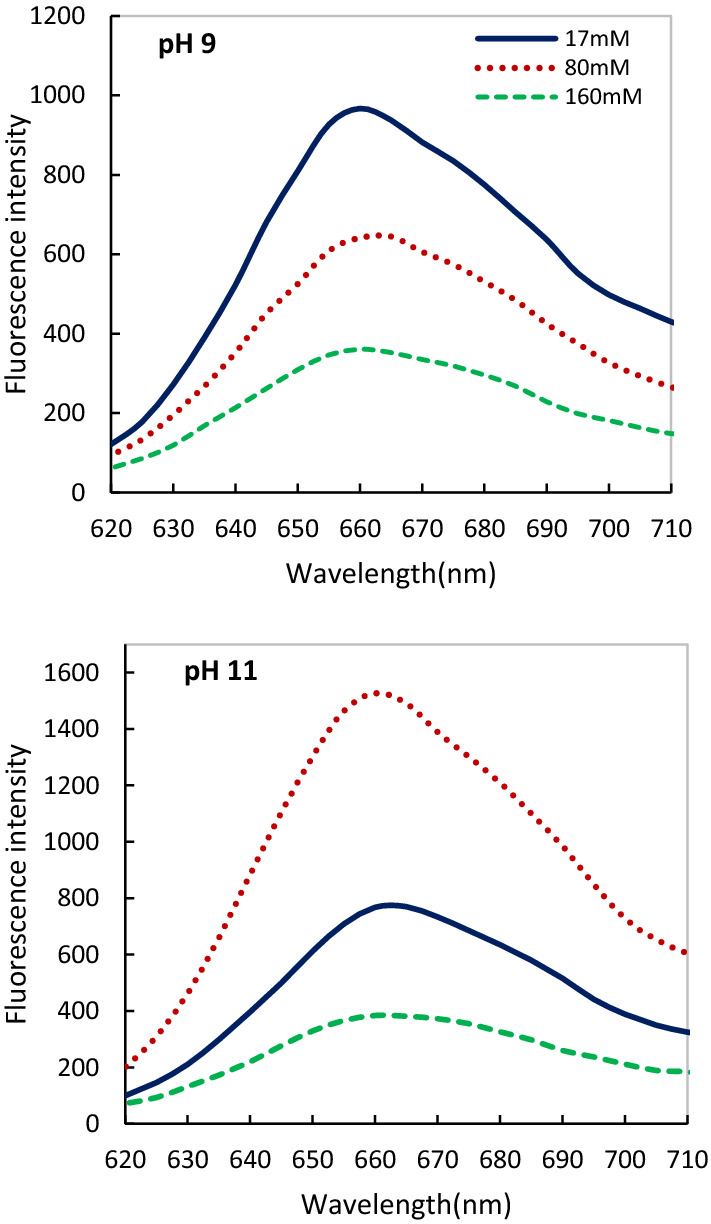
Comparison of the fluorescence intensity of *Cylindrospermum* sp. FS 64 at different salinity and pHs after 72 h of inoculation. Excitation 550 nm

We investigated the distribution of energy between phycobiliproteins and PSII spectrofluorimetrically at excitation 440 nm (chlorophyll- associated with PSII), and 550 (phycocyanin). We observed addition of 80 mM salinity was accompanied by increasing PSII (Fig. 5) and phycocyanin activity (Fig. 6) at pH 11 compared to pH 9. Although, increasing salinity (160 mM) drastically decreased the efficiency and effectiveness of PSII and PC activity at both alkaline pHs after 24 h (Figs. 5, 6). This study confirmed the high growth, biomass production, and content of the PSII and PBS (absorption spectra) under the combined effect of 80 mM salinity at pH 11.

### Spectroscopic study of a single cell by CLSM

**Fig. 7:**
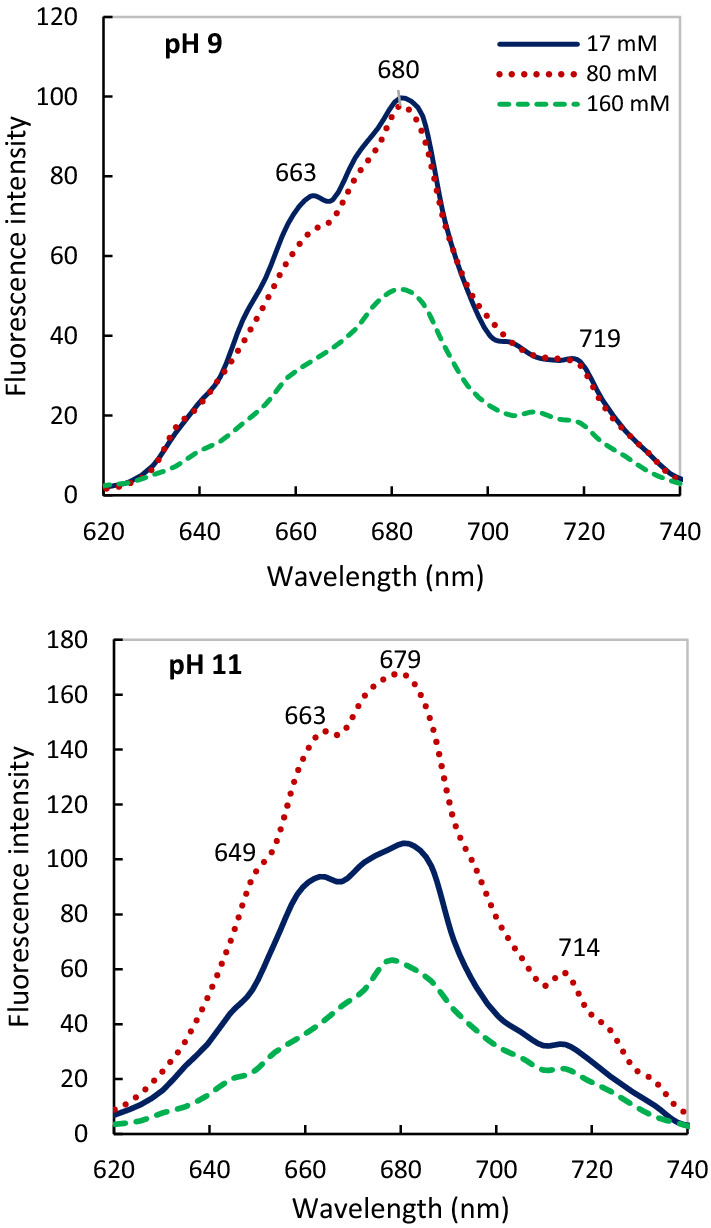
Fluorescence spectra of individual cell (Lambda scan) of *Cylindrospermum* sp. FS 64 at different salinity and pHs after 72 h of inoculation. *λ*exc = 405 nm

Investigation of single cell illustrated the physiological state of the individual, live-cell and maximum fluorescent of pigment protein which enabled us to monitor the dynamic processes of the chosen cells as spectral unmixing and all steps of the energy transfer chain (Grigoryeva and Chistyakova  2019; Shokravi and Bahavar 2021b) (Fig. 7). We observed the high fluorescence of Chl *a* (PSII) at ~ 680 nm and PSI at ~ 715 nm at vegetative cells compared to heterocyst—because of low amounts of PSII and PBS at heterocyst (heterocyte result not shown). In addition, a clear shoulder was observed around 663 nm related to the APC (Allophycocyanin) of phycobilin production, which was highest at 80 mM salinity at pH 11. Increasing salinity (160 mM) led to declined APC content at both alkaline pHs. This reduction which was saline dependent caused the demolished structure of PBS at pH 9 compared to pH 11. Chl *a* (PSII-680 nm) was the most stable pigment under all conditions, and it was higher under the combined 80 mM salinity and pH 11. No significant difference of PSII and also PSI activity was observed between 17 and 80 mM salinity after 72 h at pH 9 against pH 11. While the highest PSI activity belongs to 80 mM salinity at pH 11. Overall, our results of the single-cell study confirmed the highest growth and stability of PS and PBS depends on combined 80 mM salinity and pH 11 (Figs. 1, 5, 6).

## Discussion

Overall, our results provide important insight that combined multiple factors such as salinity, alkalinity, and the age of the cultures in laboratory conditions plays a key role in acclimatizing the growth, and photosynthesis of *Cylindrospermum* sp. FS 64. Absorption spectra and chlorophyll concentration (OD 750) methods as an overview of growth and cells activity on the culture (Schulze et al. 2011) indicated that salinity and alkalinity (combine together—pH 11 and 80 mM salinity) cannot be considered as stress to limit the growth (Borowitzka 2018) of *Cylindrospermum* sp. FS 64. Low salinity (17 and 80 mM) in heterocystous cyanobacteria (Srivastava et al. 2009) can be desired as nutrition and stimulant leads to a significant increase in growth, biomass production (Miriam et al. 2017), and photosynthesis operation (Singh et al. 2015). The elevated salinity (160 mM) at both alkaline pHs 9 and 11 led to inhibition of Chl biosynthesis (Chris et al. 2006) which resulted in a decrease in chlorophyll pigment. Besides, these findings are in line with our other study on *Nostoc* sp. UAB 206 that isolated from the Spanish paddy field (not published data). Valiente and Leganes (1990), Poza-Carrión et al. (2001), Soltani et al. (2006), Amirlatifi et al. (2018), Abbasi et al. (2019) and Shokravi and Bahavar (2021a) have indicated that the optimum pH for growth, photosynthesis and nitrogen fixation of terrestrial cyanobacteria under combined environmental factors is about 8 or 9. The response of strain to pH 11 and 80 mM salinity may depend on the high genetic plasticity (Boussiba et al. 2000) or and is an inherent characteristic (Tang and Vincent 1999) which resulted in without any requirement for an acclimation process (Vonshak and Torzillo 2007). Furthermore, we observed a strong correlation-fitting model—under the combined 80 mM salinity and pH 11 up to 96 h which confirmed the highest activity of cells under this condition. Pigment analysis of cell cultures (absorption spectra) also confirmed the results of the activity of the cells under this condition.

No accurate understanding of the mechanisms involved in pH homeostasis in cyanobacteria. Giraldez-Ruiz et al. (1997) proposed that Na, K, and Ca could be considered as part of the pH homeostatic system. Touloupakis et al. (2016) reported that many mechanisms have been suggested for pH homeostasis and the regulation of CO_2_/HCO_3_ concentration. Most carbon sources are in bicarbonate ions under severe carbon dioxide deficiency in alkaliphilic cyanobacteria (Boyd 2015). The carbon dioxide concentration mechanism (CCM) is the key process that enables them to acclimate to alkaline conditions (Klanchui et al. 2017). The operation of CCM involves a high amount of energy and naturally requires a high operation of photosynthesis—along with other needs—and high efficiency of PSI, PSII, and PBS (Mangan and Brenner 2014). Regarding the highest growth and photosynthesis at pH 11, *Cylindrospermum* sp. FS 64 have a powerful carbon dioxide concentration mechanism and flexibility to induce.

The main reasons of growth measuring is understanding the balance between photosynthesis and respiration (Nygård and Dring 2008). The oxygen liberation (a marker of PSII activity) analysis confirmed the growth results. Regardless of salinity, the maximum photosynthesis (*P*_max_) of *Cylindrospermum* sp. FS 64 was higher at pH 9 against pH 11. Addition salinity (80 mM) caused an increase in *P*_max_ (Ye and Gao 2004; Dhiab et al. 2007) at pH 11 which can be attributed to the high efficiency of water oxidation in PSII. In contrast, 80 mM salinity led to decreasing in saturating irradiance and increased shade-adapted capacity of strain at both alkaline pHs, which influenced an increase in the relative content of PSII activity and the antenna size of PS II (Inoue-Kashino et al. 2005). Briand et al. (2004) reported that *I*_*k*_ is the most reliable parameter for assessing and comparing the variable-light requirement. The high *I*_*k*_ value of *Cylindrospermum* sp. FS 64 can be ascribed to the different media, pH, and salinity used, implying an increase in energy for growth.

To better understanding PBS and PS activity and stability, we have used the fluorescence assay. The strain showed nearly 90% of PBS and PSII stability under the combination of 80 mM salinity and pH 11 after 96 h compared to pH 9. While increasing salinity (160 mM) led to demolished of the PBS and PSII structure at both alkaline pHs after 24 h. Galetović et al. (2020) reported most research focuses on PBS behavior and stability in different temperature and pH 5–7 (Antelo et al. 2008), pH 2.0, 6.5 and 8.0 (Couteau et al. 2004) and pH range of 4–9 (Leu et al. 2013). Chris et al. (2006) found a decrease in growth, chlorophyll content, carotenoid, phycocyanin, and PS II activity of *Cylindrospermum* sp. due to individual salinity as well as in combination with UV-B treatments. In addition, Srivastava et al. (2009) investigated that 150 mM salinity and pH 7.5 caused a decline in PSI, PS II, and whole chain activities in *Anabaena doliolum* after 24 h. The difference between these findings may be due to the use of various pH ranges which influences the growth, metabolism, regulation, and distribution of cyanobacteria (Jin and Kirk 2018).

During cultivation, chlorophyll content decreases due to environmental factors or dying of the part of the population (age of culture). (Schulze et al. 2011; Shokravi and Bahavar 2021a, b). Therefore, single-cell study is a new method that provide information on the dynamic behavior of each cell (Sugiura and Itoh 2012; Grigoryeva and Chistyakova  2019). By confocal laser microscopy, Ying et al. (2002), Wolf and Schüßler (2005) and Sugiura and Itoh (2012) demonstrated different fluorescence spectra of vegetative and heterocyte cells-unmixing spectra. The results of single-cell spectra support that combination 80 mM salinity and pH 11 up to 96 h led to a noticeable increase and stability in all parts of the phycobilisome and PSII activity. This stability of PSII may depend on the physical change in enzymes and binding sites in PSII and potent PSII efficiency. Reduction of PBS and PSII content indicating the degradation of their structure by increasing salinity at both alkaline pHs. We observed a decline and the shifted peak of PSI (719 nm) that is affected by the lower PC content at pH 9 compared to pH 11. Therefore, cells might accept less excitation energy when PC is reduced (Schmitt et al. 2020).

## Conclusion

In conclusion, the different methods confirmed that combining environmental factors (different alkaline pH, salinity, and time under extreme DIC limitation) can affect cyanobacterial behaviors individually or in combination and led to regulation and acclimation in short time. We observed *Cylinrospermum* sp. FS 64 acclimatized through different strategies and has developed a mechanism for the highest growth, photosystems operation, phycobilisomes activity and light-saturated photosynthetic under 80 mM salinity and pH 11. Conversely, elevated salinity was time dependent at both alkaline conditions. Several lines of evidence supported this issue. From an applied point of view, this cyanobacterium can be used in alkaline–saline paddy fields and agricultural lands as a biofertilizer, soil conditioners, and other biotechnological purposes.

## Abbreviations

APC: Allophycocyanin
Chl *a*: Chlorophyll *a*
CCM: Carbon dioxide concentrating mechanism
DIC: Dissolved inorganic carbon
PBS: Phycobilisome
PSI, PSII: Photosystems I and II
CLSM: Confocal laser scanning microscopy
